# Time-of-day of blood-feeding: effects on mosquito life history and malaria transmission

**DOI:** 10.1186/s13071-019-3513-9

**Published:** 2019-07-02

**Authors:** Aidan J. O’Donnell, Samuel S. C. Rund, Sarah E. Reece

**Affiliations:** 1 Institute of Evolutionary Biology, and Institute of Immunology and Infection Research, School of Biological Sciences, University of Edinburgh, Edinburgh, UK; 2 Department of Biological Sciences, University of Notre Dame, Notre Dame, IN 46556 USA

**Keywords:** Biological rhythm, Circadian rhythm, Fitness, Reproduction, Survival, Fecundity, *Plasmodium berghei*, *Plasmodium chabaudi*, *Anopheles stephensi*

## Abstract

**Background:**

Biological rhythms allow organisms to compartmentalise and coordinate behaviours, physiologies, and cellular processes with the predictable daily rhythms of their environment. There is increasing recognition that the biological rhythms of mosquitoes that vector parasites are important for global health. For example, whether perturbations in blood foraging rhythms as a consequence of vector control measures can undermine disease control. To address this, we explore the impacts of altered timing of blood-feeding on mosquito life history traits and malaria transmission.

**Methods:**

We present three experiments in which *Anopheles stephensi* mosquitoes were fed in the morning or evening on blood that had different qualities, including: (i) chemical-induced or (ii) *Plasmodium chabaudi* infection-induced anaemia; (iii) *Plasmodium berghei* infection but no anaemia; or (iv) stemming from hosts at different times of day. We then compared whether time-of-day variation in blood meal characteristics influences mosquito fitness proxies relating to survival and reproduction, and malaria transmission proxies.

**Results:**

Mosquito lifespan is not influenced by the time-of-day they received a blood meal, but several reproductive metrics are affected, depending on other blood characteristics. Overall, our data suggest that receiving a blood meal in the morning makes mosquitoes more likely to lay eggs, lay slightly sooner and have a larger clutch size. In keeping with previous work, *P. berghei* infection reduces mosquito lifespan and the likelihood of laying eggs, but time-of-day of blood-feeding does not impact upon these metrics nor on transmission of this parasite.

**Conclusion:**

The time-of-day of blood-feeding does not appear to have major consequences for mosquito fitness or transmission of asynchronous malaria species. If our results from a laboratory colony of mosquitoes living in benign conditions hold for wild mosquitoes, it suggests that mosquitoes have sufficient flexibility in their physiology to cope with changes in biting time induced by evading insecticide-treated bed nets. Future work should consider the impact of multiple feeding cycles and the abiotic stresses imposed by the need to forage for blood during times of day when hosts are not protected by bed nets.

## Background

Daily rhythms are a ubiquitous feature of life [1]. For example, circadian clocks are thought to enable organisms to coordinate with environmental periodicity in factors such as light/dark, humidity, UV exposure [2]. Interactions with predators, prey and hosts (in the case of parasites) also follow daily rhythms [3–5]. How daily rhythms, whether they are clock-controlled or direct responses to rhythmic environmental cues, shape, and are shaped by interactions between organisms is poorly understood. We address this by examining the consequences of daily rhythms in the interactions between vectors, their hosts, and their parasites. Specifically, we ask how the time-of-day that mosquitoes blood feed combines with the timing (phase) of rhythms in hosts and with malaria infection to shape vector fitness and disease transmission. Given reports that some mosquito populations have altered the time-of-day they bite (likely in response to the use of insecticide-treated bed nets) [6–13], exploring the consequences of perturbed blood foraging rhythms for mosquito fitness and malaria transmission is urgently required.

Mosquitoes exhibit periodicity in many fitness determining activities, including sugar feeding, the formation of mating swarms, insecticide resistance and blood-feeding [14, 15]. In keeping with this, ~ 20% of the *Anopheles gambiae* genome is expressed in patterns following daily rhythms [16]. Thus, the circadian clock enables mosquitoes to coordinate the timing of the physiological, cellular and molecular processes that underpin behaviours, with rhythms in the abiotic environment and/or other internal processes [2, 17]. For example, *Anopheline* mosquitoes are primarily night-biters [15, 18, 19] and processes associated with being active and foraging at night, including glycolysis, energy sensing and nutrient mobilization are upregulated in concert [16, 20]. Many genes, however, are not clock-regulated but still follow daily rhythms (including some *An. gambiae* odorant-binding proteins) and are driven by a direct response to light or dark [21]. Indeed in both *An. gambiae* and *Aedes aegypti*, more rhythmic genes are detected under light:dark conditions than dark:dark conditions [21, 22].

A key benefit of clock-control is that organisms can anticipate dawn/dusk and prepare in advance by up- or downregulating physiological processes. For example, processes required to cope with a blood meal are upregulated in the mosquito’s active phase (night time for *Anopheles* sp.) [14, 16]. This includes catalase and other factors used to detoxify reactive oxygen species (ROS) generated as a product of blood (heme) digestion, and members of the V-ATPase complex which drive water excretion to minimise the 3-fold increase in volume that a blood meal brings [23, 24]. Exposure to ROS increases mortality and reduces clutch size of mosquitoes [23, 25, 26]. Further, as a consequence of the detoxification of blood meal induced ROS, there is a proliferation of mosquito gut microbiota [27] which have complex interactions with parasite infection [28] that may vary in line with time-of-day a blood meal is taken. In addition to rhythms in processes associated with foraging, the activities and locations of immune effectors cycle throughout the day. For example, immune defences are upregulated during the day in diurnal insects, such as *Drosophila* [29, 30]. Whether immune defences peak at night in nocturnal mosquitoes is unknown but some immune genes implicated in interactions with malaria parasites are expressed with circadian rhythms [16]. How circadian rhythms in insect immune defences relate to protection from infection or the severity of disease is unclear. For instance, *Drosophila* challenged with *Pseudomonas aeruginosa* at night are more likely to survive the infection than those challenged in the day. However, perturbation of clock genes to generate arrhythmic mutant flies can result in both decreased survival or enhanced survival depending on the specific genes modified [31]. Further, there are complex consequences of challenging *An. stephensi* with *E. coli* or the malaria parasite *P. chabaudi* at different times of day [32, 33].

Given the potential for circadian rhythms to influence the ability of mosquitoes to cope with a blood meal and with parasites, the time-of-day that mosquitoes forage has implications for both mosquito fitness and disease transmission. These consequences are likely to be complex [14]. If feeding in the daytime means that mosquitoes are less able to cope with the osmotic and oxidative costs of blood, their fecundity and survival should suffer. Indeed, mosquitoes in poor condition as a consequence of feeding in the day may have compromised immune defence and this might explain recent observations that day-fed *An. stephensi* harbour higher densities of *P. chabaudi* than night-fed mosquitoes (although parasite rhythms also mediate this effect) [33]. Alternatively, ROS is a key player in insect immune responses and so, if day-fed mosquitoes do not manage their ROS efficiently, they may suffer collateral damage but also benefit from enhanced parasite defence. Furthermore, it is also necessary to recognise that mosquitoes feed on hosts that have their own circadian rhythms [14]. This includes rhythms in red blood cell composition and density, hematocrit, amino acid composition and immune effectors [34–39]. Thus, rhythms in the composition of mammalian blood could exacerbate (or reduce) the effects of a daytime blood meal on mosquito survival and fecundity.

Clearly, predicting the net effects of how host rhythms and vector rhythms interact to shape malaria transmission is challenging but important. Such interactions could shape the probability and intensity of infection in mosquitoes as well as mosquito population dynamics. Here, three experiments are described that probe the consequences, under a variety of scenarios, of time-of-day-specific blood-feeding for proxies estimating the fitness of mosquitoes and malaria parasites. The aims are to determine: (i) if the timing of a blood meal affects mosquito survival and fecundity; (ii) whether the effects of time-of-day are exacerbated by other characteristics of host blood or malaria infection; and (iii) the consequences of blood-feeding at different times of day for malaria transmission.

## Methods

**Fig. 1:**
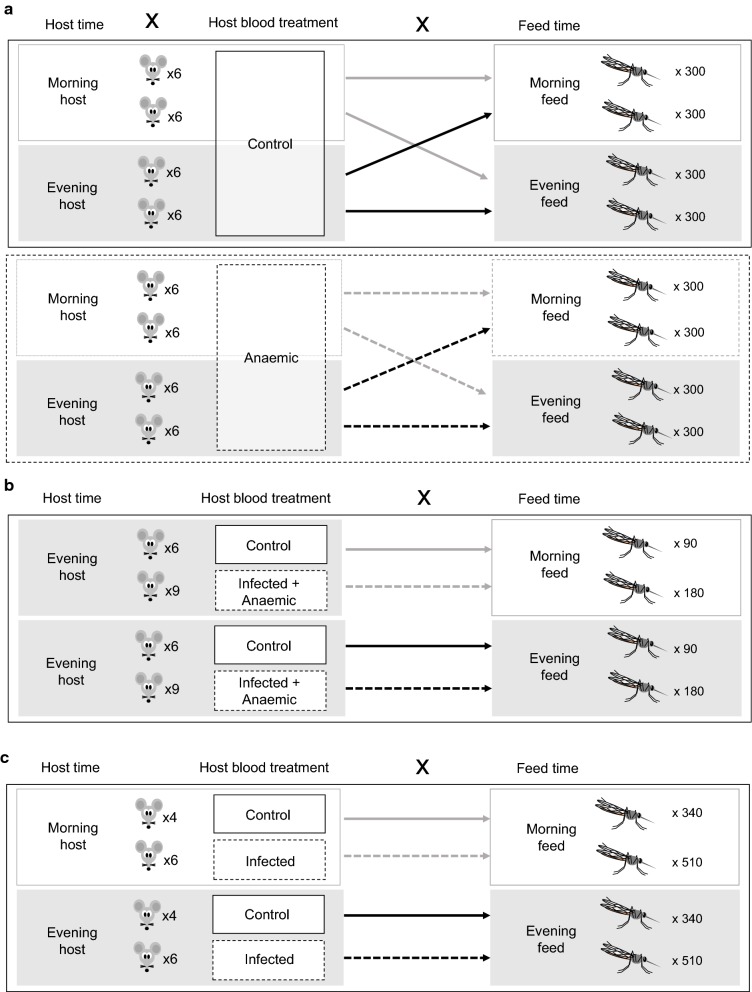
Experimental designs. For all experiments, groups of mosquitoes differ by the time-of-day they received a blood meal. Each experiment probed the effects of further perturbations of host blood: including chemically induced anaemia and host time-of-day (Experiment 1, **a**); malaria infection induced anaemia (Experiment 2, **b**); and malaria infection of mosquitoes (Experiment 3, **c**)

All experiments examine metrics of mosquito fecundity and lifespan in response to perturbing the time-of-day (morning) or (evening) that mosquitoes receive a blood meal, but differ in the following respects. The first experiment (“*blood quality and host time*”, Fig. 1a) includes the effects of both host time-of-day and feeding on blood from anaemic *versus* control mice. To further probe a role for blood quality, the second experiment (“*blood quality*”, Fig. 1b) uses a different approach to examine the effects of feeding on anaemic blood but does not consider host time-of-day. The third experiment (“*infection*,” Fig. 1c) focuses on *Plasmodium berghei* infection of mosquitoes.

### Mice

For all experiments, hosts were 10–12-week-old MF1 male mice housed at 21 °C with *ad libitum* food and drinking water supplemented with 0.05% para-aminobenzoic acid (to supplement parasite growth). Mice were housed in groups of five in either 12:12 light:dark (LD; lights on at 07:00 GMT, lights off at 19:00 GMT) or inverted dark:light photocycle (DL; lights on at 19:00 GMT, lights off at 07:00 GMT) depending on the experiment. Mice were entrained to their respective light schedules for at least 21 days prior to mosquito blood feeds. Prior to donating a blood meal, each mouse was anaesthetized (17% Dormitor, 13% Vetelar, 70% PBS administered at 4 µl/g) and then exposed to a single cage of mosquitoes.

### Mosquitoes

All *Anopheles stephensi* mosquitoes were maintained under standard insectary conditions of 27 ± 1 °C, 70% relative humidity and a 12:12 light:dark photocycle, with lights on at 07:00 GMT (ZT0) and lights off at 19:00 GMT (ZT12) (ZT0, Zeitgeber Time 0, is defined as time of lights on). Larvae were reared at a density of ~ 250 larvae per 1.5 l of distilled water. Between 12 and 14 days after hatching, pupae were transferred to emergence cages in incubators (27 ± 1 °C, 60 ± 5% relative humidity) with one-hour light ramping to simulate a dawn (starting at 07:00 GMT; ZT0) and dusk (19:00 GMT; ZT12). Mosquitoes were supplied with *ad libitum* access to 10% fructose solution supplemented with 0.05% paraminobenzoic acid. In the second experiment only, mosquitoes were treated with antibiotics (0.05% gentamicin) administered via their fructose solution 4–5 days before blood meals. For all experiments, female mosquitoes were randomly selected from 3–4 emergence cages, transferred to 2 l holding cages and starved of fructose solution for 24 h before their blood meals. Cages contained 15–85 mosquitoes (depending on the sampling regime of each experiment). Regardless of mosquito number, all mosquitoes were able to blood feed until satiated. For all feeds, each cage of females was exposed to an anaesthetized mouse for 30 min in a light setting that matched the mosquito time-of-day (i.e. morning-fed mosquitoes were fed during lights on and evening feeds were performed under dim red light). Unfed females were removed from the cages (< 5 per cage in all cases). After feeding, mosquitoes were housed in incubators at temperatures of either 20.5 or 26.0 °C (± 0.5 °C), depending on the experiment.

### Experimental designs

#### Experiment 1: blood quality and host time

**Fig. 2:**
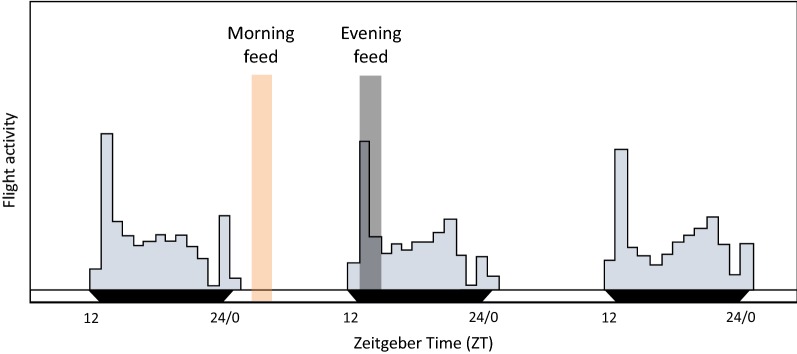
Blood feed timing. Daily flight activity of lab reared *An. stephensi* mated females (modified from [42]) showing that mosquito flight activity peaks after dusk (ZT12) with a second smaller peak before dawn (ZT0). Wild-caught mosquitoes also show this pattern, with slight variations to the size of the dusk peak depending on monsoon season [40, 41]. Shading represents timing of morning (orange) and evening (grey) blood meals in experiments 1–3. This placed the evening feeds during the mosquito’s active period and the morning feeds during the mosquito’s rest period

Mosquito cages were randomly assigned to receive a blood meal in their morning 09:00 GMT (ZT2) or evening 21:00 GMT (ZT14). These feed times are analogous to the mosquito resting period (morning) or active period (evening) as evident from wild caught and laboratory-based studies (Fig. 2, [40–42]). Within each feeding time, cages were allocated to a further four groups, based on host treatment (anaemic or control mice) and host time-of-day [morning mice (ZT2) or evening mice (ZT14)]. The availability of mice experiencing their morning or evening to feed to mosquitoes in their morning or evening was achieved by housing mice in room with LD and DL lighting schedules. This resulted in an experiment with a 2 × 2 × 2 design: eight groups varying by feed time (morning/evening), host blood treatment (anaemic/control), and host time (morning/evening) (Fig. 1a). Note, this is the only experiment that perturbs host time-of-day.

Anaemia was induced in half of the mice by intraperitoneal injection of 125 mg/kg of phenylhydrazine 3 days before feeding to mosquitoes. The control mice received a sham injection of 100 µl PBS. On the day of feeding, red blood cell (RBC) counts (×10^9^ ml^−1^) for control mice (7.48 ± 0.13 SE) were almost 2-fold higher than for phenylhydrazine treated hosts (3.80 ± 0.12 SE; *t* = 21.27, *df* = 45.67, *P* < 0.001). Each cage contained 50 mosquitoes and each of the 8 treatment groups contained 6 cages. On day 2 post-blood meal (PBM), mosquitoes were allocated to individual housing (50 ml falcon tubes with *ad libitum* access to 10% glucose solution *via* a 1.5 ml microcentrifuge tube feeder). Fecundity and mortality were tracked for 20 mosquitoes from each cage (960 total). Each female was given a 30 mm diameter Petri dish lined with filter paper and 3 mm depth of distilled water as an oviposition habitat. Mosquitoes were checked daily and if eggs were present, the egg dish was replaced. Egg bowls were photographed at the time of removal (for clutch size counts) incubated for 6 days and then photographed again so that all hatched larvae (alive and dead) could be counted (to estimate hatch rate). For all females (that did or did not lay) egg bowls were removed on day 9 PBM.

Additionally, the volume and density of blood meals were estimated for 10 randomly chosen mosquitoes from each cage (480 total) 2 hours after their blood meal. The right wing of each mosquito was photographed, and the abdomen removed and homogenised in 500 µl drabkins solution for ~ 30 min [43]. Samples were split into two 200 µl sub-samples and optical density (OD) read by a spectrophotometer at 540 nm (each mosquito was read in duplicate, and an average taken). To generate control series for each cage, 8 µl of blood was removed from each mouse used to feed mosquitoes at the time of feeding and used to generate 4 µl, 1 µl, 0.8 µl and 0.4 µl standards. Host RBC density readings (cells per µl) were also obtained at the time of feeding to calculate the RBC density of the blood meal. Wing length was obtained from the photographs, converted to mm and used to control for any potential differences in blood meal volume and density due to variation in body size (using the software package ImageJ [44]).

#### Experiment 2: blood quality

Here, instead of phenylhydrazine treatment, blood quality was perturbed by using malaria infection to generate anaemia. Mosquito cages were randomly assigned to morning 09:00 GMT (ZT2) or evening 21:00 GMT (ZT14) feed times. At each feeding time, half the cages were exposed to anaemic or (uninfected) control mice. This resulted in an experiment with a 2 × 2 design: four groups varying by the timing of their blood meal (morning/evening) and blood treatment (anaemic/control) (Fig. 1b). Note, host time-of-day was standardised by housing mice in two rooms with inverted light schedules (DL and LD), enabling both the morning- and evening-fed mosquitoes to feed on hosts experiencing their evening (host ZT14). Six cages were fed, at each time point, on control mice and nine cages, at each time point, on anaemic mice. Each cage exposed to control mice contained 15 mosquitoes and each cage exposed to anaemic mice contained 20 mosquitoes. Mortality was tracked as for Experiment 1 (but for 10 individuals per cage; 300 total) and egg dishes were provided until day 14 PBM.

All feeds occurred on mice at day 11 post-infection (PI) after infection with 1 × 10^6^ *P. chabaudi* CR parasitized RBCs or sham infection (controls; 100 µl PBS). *Plasmodium chabaudi* has a synchronous asexual cycle so donor mice were used from each room (DL and LD) to ensure that all hosts were infected with rings (i.e. parasite and host rhythms were phase matched; [45]). By day 11 PI, significant anaemia had occurred (mean RBC density × 10^9^ ml^−1^: Control = 7.88 (± 0.16 SE), anaemic = 4.44 (± 0.10 SE); *t* = 18.77, *df* = 19.28, *P* < 0.001) and hosts were mounting strong immune responses, so the parasite was not able to establish an infection in mosquitoes (parasite mating is very vulnerable to suboptimal conditions in the blood meal [46]). Thus, mosquitoes received poor quality blood as a result of a more ecologically realistic perturbation than PHZ, without the confounding effects of becoming infected themselves. This was verified by examining 10 randomly selected mosquitoes from each cage exposed to anaemic mice on day 14 PBM. Specifically, the midgut of each mosquito was dissected, stained for two minutes in 0.5% mercurochrome, washed in PBS and total oocysts per midgut were counted *via* microscopy. No oocysts were detected.

#### Experiment 3: infection

Mosquito cages were randomly assigned to four groups. Two groups received their blood meal in the morning 10:00 GMT (morning, ZT3) and the others were fed in the evening at 20:00 GMT (evening, ZT13). At each feed time, half of the cages were exposed to *Plasmodium berghei* infected mice or naïve (uninfected) control mice. This resulted in an experiment with a 2 × 2 design: four groups varying by the timing of their blood meal (morning/evening) and blood treatment (infected/uninfected) (Fig. 1c). Note that time-of-day for parasites/hosts and mosquitoes is synonymous; morning-fed mosquitoes received blood from hosts also experiencing their morning, and *vice versa* for evening-fed mosquitoes. At each time point, six cages were fed on infected mice and four fed cages on uninfected mice.

**Fig. 3:**
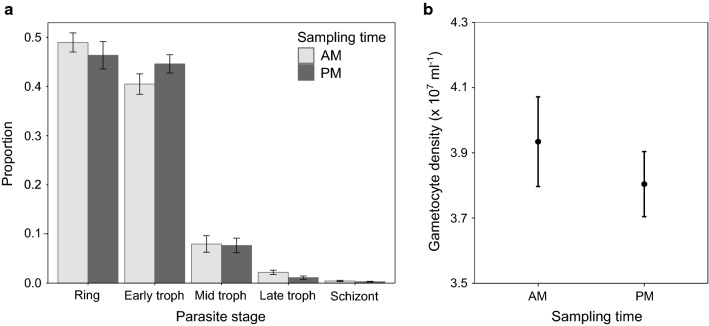
Composition of *P. berghei* parasites in mice used to infect mosquitoes in experiment 3. Shown are the means ± SE parasite stage proportions (**a**) and gametocyte density (**b**) from infected hosts used for blood meals. Donors were sampled at the time of blood meals either in the morning (AM; 10:00 GMT; ZT3) or evening (PM; 20:00 GMT; ZT13) (*n* = 6)

All feeds occurred on mice at day 6 PI after inoculation with 1 × 10^5^ *P. berghei* parasitized RBCs or sham infection (controls; 100 µl PBS). Infections (and sham injections) were staggered by 10 h to ensure that morning- and evening-fed mosquitoes were exposed to infections of the same age (144 h). *Plasmodium berghei* was chosen because its asexual cycle is asynchronous, ensuring that morning- and evening-fed mosquitoes did not receive significantly different stage distributions of asexual parasites (feed time:parasite stage; *χ*^2^_4_ = 1.28, *P* = 0.29; Fig. 3a) or gametocyte densities/ages (mean gametocyte density × 10^7^ ml^−1^: morning = 3.93 (± 0.14 SE), evening = 3.80 (± 0.1 SE); *t* = 0.77, *df* = 10, *P* = 0.46; Fig. 3b). On day 6 PI, *P. berghei* had not significantly reduced the RBC density of hosts (mean RBC density × 10^9^ ml^−1^: Control = 8.17 (± 0.08 SE), Infected = 7.85 (± 0.18 SE); *t* = 1.64, *df* = 14.71, *P* = 0.12).

At the time of feeding, each cage contained 85 mosquitoes. After the blood meal, 15 mosquitoes from cages fed on infected mice were removed (180 total) and used to monitor oocyst prevalence and density as for Experiment 2. To track mosquito fecundity and mortality, a subset of 15 randomly selected females were removed from each cage (300 total) 2 days PBM and housed individually in 200 ml cups with *ad libitum* access to 10% fructose solution. On day 3 PBM, each female was given a 30 mm diameter Petri dish lined with filter paper and 3 mm depth of distilled water as an oviposition habitat. Mosquitoes were checked daily until death, if eggs were present the egg dish was replaced (up until day 21 PBM).

### Data analysis

R version 2.6.1 [47] was used for all analyses. Model simplification was carried out by stepwise deletion of the least significant term and only minimal models are reported. Measurements made from mice at the time of feeding (red blood cell counts and parasite stage composition and densities), and time-of-day differences in infection load for mosquitoes were analysed with Studentʼs t-test. Mosquito fecundity metrics, proportion of females that laid and hatch rate, were analysed using generalised linear mixed-effects models with binomial error structures. Clutch size and blood meal measures were analysed using linear mixed effect models. In both types of linear models, identity of the mosquito cage was included as a random effect. All models met model assumptions: independence of data points, normality of residuals and homogeneity of variances (confirmed through assessing the model plots, the Shapiro–Wilk test and Bartlett’s test). Cox proportional hazard models with mosquito identity nested within cage as random effects (frailty model) were used to estimate the effects of feed time and host blood manipulations on the time taken to lay and lifespan (*coxme* package in R [48]). All Cox models model met the proportional hazards assumptions based on Schoenfeld’s residuals (evaluated using the ‘cox.zph’ function R; *P* > 0.1 for all variables). Clutch size of mosquitoes that laid and its interactions with experimental treatments was also controlled for because the data indicated considerable heterogeneity in clutch size, and trade-offs between survival and reproduction have been reported [49, 50] and may depend on resource availability, which may vary as a consequence of perturbations of blood quality. For this reason, mosquitoes that did not lay eggs were excluded from time to lay, clutch size and lifespan analyses. For all analyses, main effects and two-way interactions were investigated.

## Results

We carried out three experiments to determine how the timing of receiving a blood meal affects aspects of mosquito survival and fecundity, and whether qualities of host blood or malaria infection modulate the effects of the time-of-day that mosquitoes feed.

### Experiment 1: blood quality and host time

This experiment (Fig. 1a) recognises that hosts have circadian rhythms in blood composition and was designed to address if host time-of-day and blood quality (chemical induced anaemia) interact with mosquito feeding time-of-day to shape the following parameters (see Table 1 for a summary).

**Table 1:** Summary of statistical results for analyses in Experiment 1, Experiment 2 and Experiment 3

Fitness metric	Statistical results for each term in model
Experiment 1	
Blood meal volume	*Sig: Blood quality*
	Non-Sig: Feed time:host time; Feed time:blood quality; Host time:blood quality; Feed time; Host time
Blood meal density	*Sig: Host time:blood quality*
	Non-Sig: Feed time:host time; Feed time:blood quality; Feed time
Proportion laid	*Sig: Feed time*
	Non-Sig: Host time:blood quality; Feed time:host time; Feed time:blood quality; Host time; Blood quality
Time to lay	*Sig: Feed time*
	Non-Sig: Host time; Blood quality
Clutch size	*Sig: Feed time:blood quality*
	Non-sig: Feed time:host time; Host time:blood quality; Host time
Hatch rate	*Sig: na*
	Non-sig: Feed time:host time; Feed time:blood quality; Host time:blood quality; Feed time; Host time; Blood quality
Lifespan	*Sig: na*
	Non-sig: Host time; Feed time; Blood quality
Experiment 2	
Proportion laid	*Sig: na*
	Non-Sig: Feed time:blood quality; Blood quality; Feed time
Time to lay	*Sig: na*
	Non-Sig: Feed time:blood quality; Blood quality; Feed time
Clutch size	*Sig: Blood quality*
	Non-sig: Feed time:blood quality; Feed time
Lifespan	*Sig: na*
	Non-sig: Feed time; Blood quality
Experiment 3	
Malaria prevalence & intensity	*Sig: Infected/uninfected blood*
	Non-Sig: Feed time
Proportion laid	*Sig: Infection status*
	Non-Sig: Infection status:feed time; Feed time
Time to lay	*Sig: Infection status*
	Non-Sig: Infection status:feed time; Feed time
Clutch size	*Sig: na*
	Non-sig: Infection status:feed time; Feed time; Infection status
Lifespan	*Sig: Infection status*
	Non-sig: Feed time

*Notes*: Terms that significantly affected the mosquito fitness metric in question are highlighted in italics. Interactions between terms are indicated by ‘:’ and main effects are not included for terms involved in significant interactions

#### Blood meal: volume and density

**Fig. 4:**
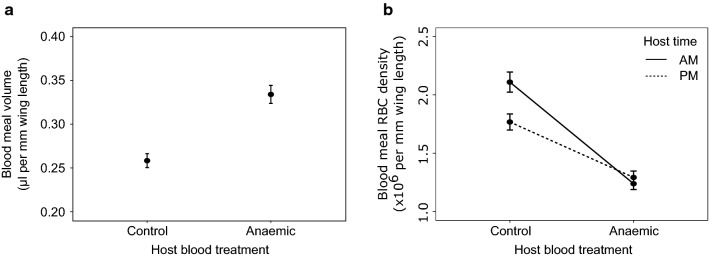
Blood meal volume (**a**) and blood meal RBC density (**b**) in response to perturbations of host blood quality and host time-of-day (Experiment 1). Mean ± SE, adjusted for body size. AM indicates morning for hosts and parasites, at 09:00 GMT (ZT 2) and PM indicates 21:00 GMT (ZT 14). Anaemia was induced in hosts by phenylhydrazine (125 mg/kg injected intraperitoneally)

There was no significant effect of feed time (*χ*^2^_7_ = 1.02, *P* = 0.31), host time (*χ*^2^_5_ = 0.01, *P* = 0.91), or their interaction (*χ*^2^_9_ = 0.40, *P* = 0.53) on the volume of the blood meal. The effect of host blood quality was not significantly influenced by interactions with feed time (*χ*^2^_8_ = 0.75, *P* = 0.39) or host time (*χ*^2^_6_ = 2.29, *P* = 0.13). However, mosquitoes that fed on anaemic hosts took up a greater volume of blood than those that fed on control hosts (mean ± SE blood meal volume (µl) per mm wing length: control = 0.26 ± 0.01), anaemic = 0.33 ± 0.01; *χ*^2^_4_ = 17.90, *P* < 0.0001; Fig. 4a). There was also a borderline significant interaction between host time and host blood quality on the RBC density of the blood meal (*χ*^2^_6_ = 4.30, *P* = 0.038; Fig. 4b). Specifically, mosquitoes that fed on control hosts consumed more RBCs than those that fed on anaemic hosts, especially when fed on hosts that experienced their morning (mean ± SE × 10^6^; control hosts: morning = 2.11 ± 0.94, evening = 1.77 ± 0.75; anaemic hosts: morning = 1.24 ± 0.54, evening = 1.29 ± 0.60). There was no significant effect of feed time (*χ*^2^_7_ = 0.66, *P* = 0.42), nor its interactions with host blood quality (*χ*^2^_9_ = 0.65, *P* = 0.42) or host time (*χ*^2^_8_ = 3.25, *P* = 0.07) on the RBC of the blood meal.

#### Reproduction: proportion laid

**Fig. 5:**
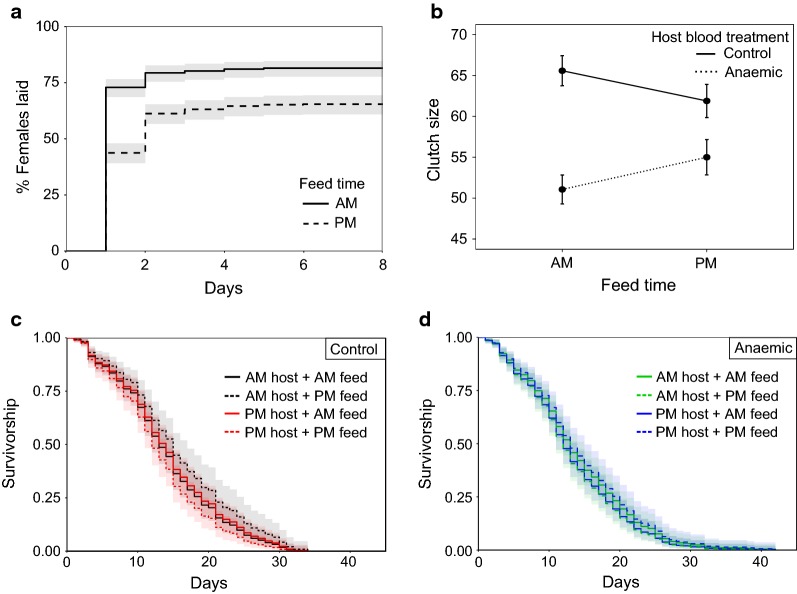
Mosquito reproduction and survival in response to perturbations of host blood quality and host time-of-day (Experiment 1). Shown are Kaplan–Meier curves for the time taken to lay (**a**), mean ± SE clutch size (**b**), and Kaplan–Meier curves for mosquito survival (**c**, **d**). AM indicates a morning feed at 09:00 GMT (ZT 2) and PM an evening feed at 21:00 GMT (ZT 14). Mosquitoes that did not lay are omitted from **b**, **c** and **d**. Due to the negative effect of clutch size on survival Kaplan–Meier curves for survival (**c** and **d**) were estimated based on median clutch size of 65. Lines represent time to lay event (**a**) and survival estimates with 95% confidence intervals in shading (**c**, **d**). Anaemia was induced in hosts by phenylhydrazine (125 mg/kg injected intraperitoneally)

Neither host blood quality (*χ*^2^_5_ = 1.60, *P* = 0.21), host time (*χ*^2^_4_ = 2.44, *P* = 0.12), or their interaction (*χ*^2^_8_ = 0.02, *P* = 0.89) significantly affected the probability each mosquito laid. However, feed time did matter, with mosquitoes that fed in the morning more likely to lay than those that fed in the evening (mean ± SE proportion of females that laid: morning = 0.82 ± 0.02, evening = 0.65 ± 0.02; *χ*^2^_3_ = 27.56, *P* < 0.0001; Fig. 5a). However, feed time did not significantly interact with either host blood quality (*χ*^2^_6_ = 0.41, *P* = 0.52) or host time (*χ*^2^_7_ = 0.05, *P* = 0.83).

#### Reproduction: time to lay

For mosquitoes that laid, neither host blood quality (*z* = 0.73, *P* = 0.47) or host time (*z* = 0.95, *P* = 0.34) influenced the time it took mosquitoes to lay eggs. Feed time did have an effect with mosquitoes that fed in the morning laying sooner than those that fed in the evening (mean ± SE days taken to lay since egg bowls were provided: morning = 1.15 ± 0.03, evening = 1.44 ± 0.04; evening:morning HR = 0.64 ± 0.08, *z* = − 5.35, *P* < 0.001; Fig. 5a).

#### Reproduction: clutch size

Clutch size was shaped by a borderline interaction between feed time and host blood quality (*χ*^2^_6_ = 4.13, *P* = 0.042; Fig. 5b). Mosquitoes fed on control mice had higher clutch sizes than those that fed on anaemic hosts, and this difference was greatest when mosquitoes fed in the morning (mean ± SE clutch size: morning-fed: control hosts = 65.57 ± 1.77, anaemic hosts = 51.06 ± 1.70; evening-fed: control hosts = 61.88 ± 1.97, anaemic hosts = 55 ± 2.09). There was also a non-significant tendency for mosquitoes that fed on hosts experiencing their morning to have higher clutch size (mean ± SE clutch size: morning hosts = 56.36 ± 1.37, evening hosts = 59.99 ± 1.34; *χ*^2^_7_ = 3.70, *P* = 0.054). However, this trend for an effect of host time was not modulated by feed time (*χ*^2^_8_ = 3.06, *P* = 0.08) or host blood quality (*χ*^2^_9_ = 0.03, *P* = 0.87).

#### Reproduction: hatch rate

Neither host blood quality (*χ*^2^_4_ = 0.12, *P* = 0.73) or host time (*χ*^2^_6_ = 0.02, *P* = 0.89) or their interaction (*χ*^2^_8_ = 0.10, *P* = 0.75) influenced egg hatch rate. Likewise, there was no significant influence of feed time (*χ*^2^_3_ = 1.31, *P* = 0.25) and its interactions with host blood quality (*χ*^2^_5_ = 2.12, *P* = 0.15) and host time (*χ*^2^_7_ = 0.40, *P* = 0.53). Mean hatch rate for all clutches was 0.69 (± 0.01 SE).

#### Lifespan

For mosquitoes that laid, neither host blood quality (*z* = 0.28, *P* = 0.78), host time (*z* = 0.75, *P* = 0.45) or feed time (*z* = − 0.10, *P* = 0.92) significantly influenced mortality rate (Fig. 5c–d). Clutch size was negatively associated with survival hazard (clutch HR = 0.996, *z* = − 2.54, *P* = 0.011), with smaller clutches (< 60 eggs) associated with a greater hazard than larger clutches (> 60 eggs). The median lifespan for all mosquitoes (that laid) was 13 days post-blood meal.

### Experiment 2: blood quality

Experiment 1 suggested that blood quality and mosquito time-of-day of feeding shaped some mosquito reproductive measures (tendency to lay and clutch size). Experiment 2 (Fig. 1b) further investigated time-of-day of feeding and blood quality by using *P. chabaudi* malaria infection to generate anaemia (see Table 1 for a summary). Host time-of-day was not investigated further because Experiment 1 revealed that it did not significantly shape mosquito reproduction or lifespan (host time-of-day only remained in an interaction with borderline significance for blood meal density).

#### Reproduction: proportion laid

**Fig. 6:**
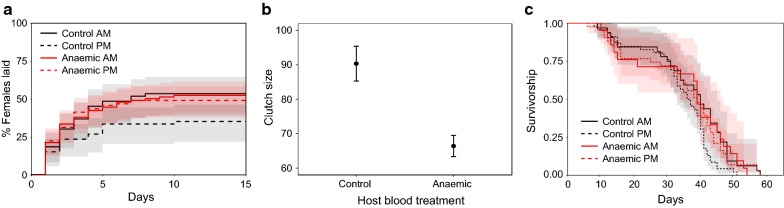
Mosquito reproduction and survival in response to perturbations of host blood quality (Experiment 2). Shown are Kaplan–Meier curves for the time taken to lay (**a**), mean ± SE clutch size (**b**), and Kaplan–Meier curves for survival (**c**). Mosquitoes that did not lay are omitted from **b** and **c**. Lines represent time to lay event (**a**) and survival estimates with 95% confidence intervals in shading (**c**). AM indicates a blood feed in the morning at 09:00 GMT (ZT 2) and PM a feed in the evening at 21:00 GMT (ZT 14). Anaemia was induced by *P. chabaudi* malaria infection, but the parasites were not infectious to mosquitoes

Neither host blood quality (*χ*^2^_4_ = 0.73, *P* = 0.39), feed time (*χ*^2^_3_ = 1.59, *P* = 0.21) nor their interaction (*χ*^2^_5_ = 1.07, *P* = 0.30) significantly influenced the proportion of females that laid (Fig. 6a). The mean proportion of females that laid per cage was 0.48 (± 0.04 SE).

#### Reproduction: time to lay

For mosquitoes that laid, neither host blood treatment (*z* = 0.52, *P* = 0.60), feed time (*z* = 0.99, *P* = 0.32) nor their interaction (*z* = 0.74, *P* = 0.46) significantly affected the time taken to lay (Fig. 6a). The average number of days to lay since eggs bowls were provided was 2.63 (± 0.17 SE).

#### Reproduction: clutch size

Host blood quality significantly affected clutch size, with mosquitoes fed on control blood laying larger clutches than mosquitoes that received anaemic blood (mean ± SE clutch size: control hosts = 90.36 ± 5.06, anaemic hosts = 66.41 ± 3.06; *χ*^2^_4_ = 8.62, *P* = 0.003; Fig. 6b). Feed time did not influence clutch size (*χ*^2^_5_ = 1.84, *P* = 0.17) or modulate the effect of blood quality (*χ*^2^_6_ = 1.81, *P* = 0.18).

#### Lifespan

For mosquitoes that laid, neither host blood quality (*z* = 1.05, *P* = 0.29), feed time (*z* = − 0.98, *P* = 0.33) or clutch size (*z* = − 1.81, *P* = 0.07) influenced mortality rates (Fig. 6c). The median lifespan was 39 days post-blood meal.

### Experiment 3: infection

Having investigated blood quality and host time-of-day in the previous experiments, we switched focus to consider the effects of malaria infection by feeding mosquitoes on blood with infectious *P. berghei* parasites at different times of day (Fig. 1c and Table 1). In addition to the effects on mosquito reproduction and lifespan, the performance of parasites was also examined.

#### Parasites

**Fig. 7:**
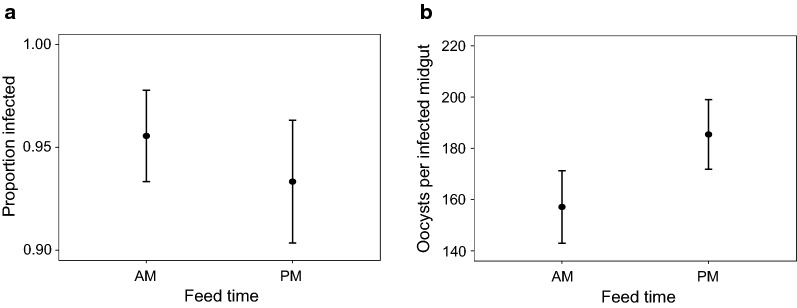
Mosquito parasite burden and intensity in response to perturbations to blood meal time (Experiment 3). Shown are the mean ± SE proportion of mosquitoes infected with *P. berghei* parasites (**a**) and mean ± SE oocyst burden per infected midgut (**b**). AM indicates a blood feed in the morning at 10:00 GMT (ZT 3) and PM a feed in the evening at 20:00 GMT (ZT 13)

No mosquitoes that fed on control hosts became infected but 94 (± 2%) mosquitoes that fed on infected hosts contained oocysts. Within mosquitoes fed on infected hosts, feed time did not influence infection prevalence (*t*_(10)_ = 0.598, *P* = 0.56; Fig. 7a) or the intensity of infection (mean ± SE oocysts = 171.3 ± 9.84; *t*_(178)_ = − 1.442, *P* = 0.15; Fig. 7b).

#### Reproduction: proportion laid

**Fig. 8:**
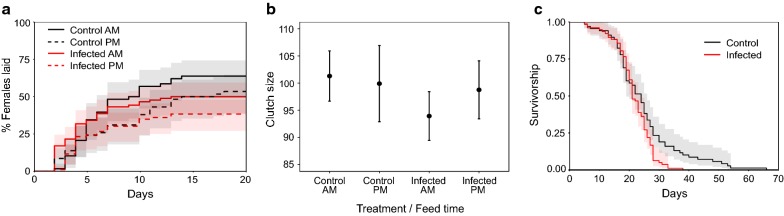
Mosquito reproduction and survival in response to malaria infection (Experiment 3). Shown are Kaplan–Meier curves for the time taken to lay (**a**), mean ± SE clutch size (**b**), and Kaplan–Meier curves for mosquito survival (**c**). Mosquitoes that did not lay are omitted from **b** and **c**. Lines represent time to lay event (**a**) and survival estimates with 95% confidence intervals in shading (**c**). AM indicates a blood feed in the morning at 10:00 GMT (ZT 3) and PM a feed in the evening at 20:00 GMT (ZT 13). Approximately 95% of infected mosquitoes were parasitized with *P. berghei* whereas control mosquitoes fed on uninfected, healthy hosts

A significantly greater proportion of uninfected than infected mosquitoes laid eggs (mean ± SE proportion laid: uninfected = 0.59 ± 0.04, infected = 0.44 ± 0.04; *χ*^2^_3_ = 5.44, *P* = 0.0197; Fig. 8a). The influence of infection was not modulated by feed time (*χ*^2^_5_ = 0.009, *P* = 0.93) but there was a trend in which mosquitoes fed in the morning were more likely to lay (mean ± SE morning = 0.56 ± 0.04, evening = 0.44 ± 0.05; *χ*^2^_4_ = 3.66, *P* = 0.056; Fig. 8a).

#### Reproduction: time to lay

For mosquitoes that laid, those that were infected laid two days sooner than uninfected individuals and at any time point, were ~ 70% more likely to lay (mean ± SE days taken to lay since egg bowls were provided: uninfected = 6.88 ± 0.48, infected = 5.05 ± 0.35; infected:uninfected Hazard Ratio (HR) = 1.71 (± 0.171, *z* = 3.12, *P* = 0.002; Fig. 8a). Neither the interaction between infection status and feed time (*z* = 0.28, *P* = 0.78) nor feed time alone (*z* = − 1.82, *P* = 0.07) influenced time to lay.

#### Reproduction: clutch size

Neither host blood treatment (*χ*^2^_4_ = 0.59, *P* = 0.44), feed time (*χ*^2^_5_ = 0.07, *P* = 0.79) nor their interaction (*χ*^2^_6_ = 0.30, *P* = 0.59) significantly influenced clutch size (Fig. 8b). Females laid an average of 98 (± 2.6 SE) eggs per clutch.

#### Lifespan

For mosquitoes that laid, infection had a negative effect on lifespan with infected mosquitoes dying sooner than uninfected mosquitoes (median lifespan: infected = 21 days, uninfected = 24 days). Further, infected mosquitoes had a ~ 50% higher overall hazard of dying (infected:uninfected HR = 1.53 (± 0.178 SE), *z* = 2.4, *P* = 0.016; Fig. 8c). Neither feed time (*z* = − 0.98, *P* = 0.33) nor clutch size (*z* = − 0.56, *P* = 0.57) influenced lifespan.

## Discussion

Here, we examine whether the fitness of female *An. stephensi* mosquitoes is affected by the time-of-day they receive a blood meal, either directly or through interactions with perturbations of blood quality and malaria infection. Specifically, we compared mosquitoes fed on control or anaemic hosts (using two different manipulations of anaemia) in which host time-of-day also varied, and whether mosquitoes were uninfected or infected with *P. berghei* malaria. The results of the three experiments are summarised in Table 2 from the perspective of the mosquitoes’ time-of-day of feeding. Overall, we found few effects of time-of-day of feeding. First, morning-fed mosquitoes appeared > 25% more likely to lay than evening-fed (Figs. 5a, 6a, 8a). Secondly, in response to chemically induced anaemia, morning-fed mosquitoes laid 0.3 of a day sooner (Fig. 5a) and produced ~ 15 (30%) more eggs than evening-fed mosquitoes (Fig. 5b). However, time-of-day of feeding did not substantially influence longevity of mosquitoes or the prevalence and intensity of *P. berghei* infection (Fig. 7). We also found mixed results for the other variables manipulated in the experiments. Host time-of-day did not influence any of the mosquito fitness metrics we measured. The effects of blood quality were similar across both of the experiments in which it was perturbed (Experiments 1 and 2); only clutch size varied in response, in which mosquitoes fed on anaemic blood laid ~ 20 (~ 22%) fewer eggs (Figs. 5b, 6b). Infection status also correlated with fitness metrics; infected mosquitoes had shorter lifespans (21 days post-blood meal for infected *vs* 24 days for uninfected; Fig. 8c), were less likely to lay eggs (44% *vs* 60% laid) and laid sooner (~2 days) than uninfected mosquitoes (Fig. 8a).

**Table 2:** Summary of statistically significant effects of the time-of-day that mosquitoes blood feed on life history traits

	Experiment 1: “Blood quality & host time”	Experiment 2: “Blood quality”	Experiment 3: “Infection”
Infection prevalence & intensity	na	na	No effect of feed time
Proportion laid	Morning-fed are ~ 26% more likely to lay than evening-fed	No effect of feed time	Morning-fed are possibly ~ 27% more likely to lay than evening fed (*P* = 0.056)
Time to lay	Morning-fed are ~ 1.5 times more likely to lay each day than evening-fed	No effect of feed time	No effect of feed time
Clutch size	Higher if fed on control (non-anaemic) blood in the morning (30% more eggs)	No effect of feed time	No effect of feed time
Hatch rate	No effect of feed time	na	na
Blood meal volume & density	No effect of feed time	na	na
Survival	No effect of feed time	No effect of feed time	No effect of feed time

*Abbreviation*: na, not available

We expected that mosquitoes receiving a blood meal at an unexpected time-of-day (i.e. morning) would experience fitness costs in the form of reduced lifespan and/or loss of reproduction. However, we found no effect on lifespan and the effects on reproduction were not consistent with costs; a higher probability of laying eggs and a modest increase in fecundity appear to be fitness benefits from morning feeding (Table 2). Laying sooner may be a fitness cost if it results in poor quality eggs or trade-off against immune defence [51]. However, we found no evidence of a quantity-quality reproductive trade-off because eggs from females in all groups (Experiment 1) hatched at a similar rate. In Experiments 2 and 3 we saw little difference between morning- and evening-fed mosquitoes in the time taken to lay despite the 10-hour ‘head-start’ of morning-fed mosquitoes. If egg maturation takes a fixed window of time since feeding, this suggests morning-fed mosquitoes are deliberately delaying their oviposition or waiting until the next ‘gate’ to oviposit if oviposition is clock-controlled. Mark-recapture studies with wild *An. farauti* show that an earlier feed time is associated with irregularities in oviposition cycle, sometimes lengthening or shortening by a day [52–54]. This demonstrates flexibility in the day of oviposition post-blood meal, but whether there is additional flexibility for time-of-day requires further investigation [55, 56]. Given daily mortality risk for mosquitoes (estimated to be around 10% for *An. gambiae* [57]), intuition suggests it would be adaptive to lay as soon as they are able.

A lack of costs of morning feeding could have several non-mutually exclusive explanations. First, costs were expected because the expression of numerous genes involved in processes required to neutralise the ROS produced by blood digestion is rhythmic [16]. However, transcriptional circadian phases do not always reflect protein abundance rhythms [58, 59]. Nine of the 12 V-ATPase subunits of the vesicular type H+ ATPase (V-ATPase), which is associated with maintaining osmotic balance during the increase in volume resulting from a blood meal, are rhythmic at the protein level (peaking at dusk in *An. gambiae* [16]). This has led to the suggestion that water excretion is compromised in mosquitoes feeding in the daytime and so, they should compensate by taking smaller blood meals [14]. However, we found no evidence of feeding time-of-day affecting blood meal volume or density. Secondly, immune responses are suggested to be timed to defend against pathogens acquired during foraging [60]. However, for mosquitoes, there may be an acute need for immune control of the proliferation of gut microbiota that expand upon an influx of blood [61]. ROS favours pathogen defence and a combination of digestion-related and immune-related ROS might erode rhythmicity in ROS levels, or defences may be upregulated as a direct response to feeding, rather than in a time-of-day dependent manner. Thirdly, when only comparing two time points on a symmetrical curve, there is a risk of picking the same intercept as the curve ascends and descends (“shoulder problem”). However, this is unlikely to be the case in our experiments because mosquitoes were in their rest phase in morning feeds and their active phase in evening feeds [42]. Fourthly, if feeding at the wrong time-of-day has only minor negative fitness consequences, manipulating feeding time-of-day over multiple blood-feeding and oviposition cycles might be required to detect costs, or keeping mosquitoes in a more stressful and ecologically realistic manner.

Many nutrients and amino acids in the blood that are essential to mosquito egg development (e.g. isoleucine) [62] exhibit circadian periodicity [34–36] but we found no evidence that host time-of-day matters for mosquitoes feeding on either healthy or anaemic mice. Mice take their largest meal around lights off, and so, by carrying out feeds on mice several hours into their active *versus* rest phases the difference in blood meal composition due to metabolic processes should have been considerable. Perhaps these factors are not limiting at any point in their rhythms, especially for mosquitoes receiving blood from well-fed laboratory mice. Further work could consider investigating the role host time-of-day in more dramatic manipulations of blood composition, for example, during infection and under food-limited conditions.

Our perturbations of anaemia did affect mosquito reproduction; clutch size was reduced in mosquitoes feeding on anaemic blood. Inducing anaemia with phenylhydrazine causes oxidative damage to red blood cells which are then cleared from circulation [63]. ROS damages mosquitoes [23, 25, 26], but by feeding mosquitoes three days after phenylhydrazine administration, the ROS it causes should have been neutralised. Thus, the main difference between blood from control and phenylhydrazine-treated mice is the age structure and density of RBC. Our data suggest that mosquitoes take up a larger volume of blood from phenylhydrazine-treated mice (perhaps facilitated by lower viscosity of anaemic blood [64]), but that this does not fully compensate and equalise blood meal RBC densities to those from feeds on control mice (Fig. 4). There may be additional differences in blood quality between chemical- and infection-induced anaemia. However, given their similar impacts, the ability to garner fewer resources from anaemic blood could explain the reduction in clutch size we observed. This is supported by previously revealed positive correlations between haematin content of blood and clutch size [65, 66]. Additionally, we found that hatch rate is a decreasing function with lay day (*χ*^2^_5_ = 12.58, *P* < 0.001) but only in those mosquitoes that fed on anaemic hosts. This result is similar to that reported in infected mosquitoes [67] suggesting that this result may be an effect of blood quality rather than parasite infection.

Our results contrast with recent work showing that mosquitoesʼ blood-feeding in the daytime are more likely to become infected after feeding on *P. chabaudi* infected mice, although *P. chabaudi* oocyst burdens did not differ between feed times [33]. Compared to *P. berghei*, *P. chabaudi* generally transmits with far lower prevalence and burden, which may facilitate detection of subtle time-of-day effects. An alternative possibility is that mosquito time-of-day effects are driven by an interaction with parasite time-of-day and so, are only observed in infections with synchronously developing parasites such as *P. chabaudi* (in which a specific age of gametocytes is present in blood meals), or in asynchronous species such as *P. relictum* in which parasite abundance in the blood (rather than age) is rhythmic [68]. In contrast to the effects of our other perturbations in the experiments presented here, we found negative effects of infection on lifespan. Costs of malaria infection on mosquito lifespan have been observed in other malaria model systems (reviewed in [69]) as well as an advancement of egg laying [67]. The advanced laying of infected mosquitoes may be a form of terminal investment because organisms with low survival prospects rush to reproduce before dying [70–73]. If our mosquitoes adopted terminal investment, it is necessary to explain why uninfected mosquitoes do not benefit from early reproduction. This could be because advancing reproduction also results in reduced clutch size (but we did not observe this), lower hatch rate (Experiment 1 suggests this does not occur either), trade-offs against anti-parasite immune responses, or reduces the probability or size of future clutches [74]. Alternatively, mosquitoes may restrict essential lipid resources available to parasites by allocating them to eggs as quickly as possible [75] or since mounting an immune response is costly to fecundity, laying early may be a compromise for both fecundity and survival [76].

## Conclusions

In summary, we found that taking a blood meal in the morning compared to the evening has no, or minor negative, effects on the fitness of mosquitoes, nor impacts upon on *P. berghei* malaria infection. If our results from a laboratory colony of mosquitoes living in benign conditions hold for wild mosquitoes, it suggests that mosquitoes have sufficient flexibility in their physiology to cope with changes in biting time induced by evading insecticide-treated bed nets. Future work should consider the impact of multiple feeding cycles and the abiotic stresses imposed by the need to forage for blood when hosts are not protected by bed nets.

## Data Availability

The datasets supporting the conclusions of this article are available in the Edinburgh DataShare repository, 10.7488/ds/2485.
